# Boosting efferocytosis in alveolar space using BCG vaccine to protect host against influenza pneumonia

**DOI:** 10.1371/journal.pone.0180143

**Published:** 2017-07-07

**Authors:** Sanjay Mukherjee, Renuka Subramaniam, Han Chen, Anthony Smith, Shiva Keshava, Homayoun Shams

**Affiliations:** 1 Center for Pulmonary and Infectious Diseases Control (CPIDC), University of Texas Health Science Center at Tyler, Tyler, TX, United States of America; 2 Department of Cellular and Molecular Biology, University of Texas Health Science Center at Tyler, Tyler, TX, United States of America; Central Michigan University College of Medicine, UNITED STATES

## Abstract

Efferocytosis by alveolar phagocytes (APs) is pivotal in maintenance of lung homeostasis. Increased efferocytosis by APs results in protection against lethal acute lung injury due to pulmonary infections whereas defective efferocytosis by APs results in chronic lung inflammation. In this report, we show that pulmonary delivery of Bacillus Calmette-Guerin (BCG) significantly enhances efferocytosis by APs. Increased efferocytosis by APs maintains lung homeostasis and protects mice against lethal influenza pneumonia. Intranasally treated wild type C57Bl/6 (WT) mice with BCG showed significant increase in APs efferocytosis in vivo compared to their PBS-treated counterparts. All BCG-treated WT mice survived lethal influenza A virus (IAV) infection whereas all PBS-treated mice succumbed. BCG-induced resistance was abrogated by depleting AP prior to IAV infection. BCG treatment increased uptake, and digestion/removal of apoptotic cells by APs. BCG significantly increased the expression of TIM4 on APs and increased expression of Rab5 and Rab7. We demonstrated that increased efferocytosis by APs through pulmonary delivery of BCG initiated rapid clearance of apoptotic cells from the alveolar space, maintained lung homeostasis, reduced inflammation and protected host against lethal IAV pneumonia.

## Introduction

Several mechanisms are involved in lung homeostasis such as mucociliary clearance and phagocytosis. Alveolar phagocytes(APs) consists of mostly alveolar macrophages (AMs), recruited monocytes and dendritic cells (DCs) are the most prominent phagocytes in the lung and play pivotal roles in uptake, digestion and removal of dead and apoptotic cells, cell debris, pathogens and inhaled particles. Defective phagocytosis by APs results in chronic inflammation in the lungs and significantly increases the likelihood of developing chronic obstructive pulmonary disease (COPD), lung injury and cancer [1–4]. Alveolar macrophages (AM)s from patients with airway diseases such as COPD, asthma, and cystic fibrosis have impaired phagocytic function [5–7].

Bacillus Calmette-Guerin (BCG) vaccine is a live attenuated *Mycobacterium bovis* and the only available anti-tuberculosis vaccine. BCG has been used for more than 90 years with overwhelming safety records [8] both as an anti-tuberculosis vaccine and more importantly, as an immunotherapeutic agent to treat other diseases. It has been attributed to reduced leprosy cases in the past several decades, used to treat melanoma cases, and through intravesical delivery, BCG is part of standard regimen to treat and prevent the recurrence of superficial bladder tumors [9–11]. BCG also confers a non-specific protection against influenza infections in mice [12]. Substantial evidences for nonspecific beneficial effects of BCG vaccination in humans have been provided by a randomized clinical trial [13]. BCG increases non-specific protection against other diseases mostly through enhancement of macrophage functions. Presence of BCG has been shown to increase the recruitment of macrophages as well as macrophage activation [14]. In this report we tested the role of BCG on efferocytosis by APs and mechanisms by which it protects against lethal influenza pneumonia.

Our data show that pulmonary delivery of BCG significantly enhances efferocytosis by APs. Increased efferocytosis by APs maintains lung homeostasis and radically improves the outcome of acute pneumonia. Wild type C57Bl/6 (WT) mice were intranasally immunized with BCG and showed significant increase in APs efferocytosis in vivo compared to their PBS-treated counterparts. All BCG-immunized WT mice survived lethal influenza A virus (IAV) infection whereas all PBS-treated mice succumbed to IAV. BCG-induced resistance was abrogated by depleting AP prior to IAV infection. Our results confirmed that pulmonary delivery of BCG increased efferocytosis by APs that resulted in rapid clearance of apoptotic cells from the alveolar space, and consequently reduced inflammation and lung injury. We identified the mechanisms that mediated this protection and developed a novel intervention for increasing the efferocytic activities by APs.

## Materials & methods

### Mice

Six-eight week old wild typeC57BL/6 (WT) mice purchased from Jackson laboratory (ME) were used in all experiments. All the experimental protocols were approved by the Institutional Animal Care and Use Committee (IACUC) at the University of Texas Health Science Center at Tyler.

### BCG vaccination

Unless otherwise stated,WT mice were vaccinated with TICE strain of BCG [4x10^4^ colony forming units (CFU), Organon USA Inc. Roseland, NJ 07068] intranasally (IN). Viability and bacterial load was determined by plating inoculum or lung homogenates on 7H10 medium. In all IN BCG immunizations, 50 μl inoculum was delivered under general anesthesia using ketamine/xylazine intraperitoneally (IP).

### Influenza A virus (IAV) infection

Mouse-adapted influenza virus A/Puerto Rico/8/34 (PR8) (H1N1) strain (CharlesRiver,Wilmington,MA) was used in all experiments. Mice were inoculated with 50 μl of PBS containing the PR8 strain IN under general anesthesia using IP injection of Ketamine/Xylazine. The LD50 (Lethal dose 50) of the virus strain was determined by limiting dilution[15].

### Mouse lung homogenate preparation

Mice were euthanized and their lungs were removed aseptically and homogenized in a cell strainer until no masses were present. Homogenates were then immediately stored at -80°C.

### Quantification of virus

Madin-Darby Canine Kidney (MDCK) cells were cultured in complete RPMI 1640. Ten-fold dilutions of lung homogenates or samples were made in medium with 1μg/ml TPCK and added onto MDCK cells. Plates were swirled for virus absorption, supernatant was removed and fresh medium was added. After 72 hours, the virus titer was quantified using the hemagglutination assay[15,16]. Culture supernatants (50 μL) were transferred into 96 well plates containing 0.5% chicken red blood cells (RBC) and incubated for 45 minutes at room temperature. Hemagglutination of chicken RBC was recorded and the median tissue culture infective dose (TCID50) was calculated by the Spearman-Karber formula.

### Bronchoalveolar lavage (BAL) collection

Mice were euthanized, the ribcage was severed, and a tracheotomy was performed. Lung perfusion was carried out to remove blood from pulmonary vasculatures. Mouse lungs were perfused three times with fresh 1X PBS using 22G catheters. BALs were centrifuged at 1500 RPM for 10 minutes. Over 99% of BALs were alveolar macrophages (AM) as identified by staining and morphometry by independent hematology technicians.

### Apoptotic cell preparation

Chemically induced apoptotic mouse lung epithelial (MLE) cells were used for all the efferocytosis assays. MLE cells were cultured for 24 h in 6-well plates. Uniform apoptosis was induced in MLE cells (ATCC^®^ CRL-2110^™^) by incubating them with 2 μM staurosporine[17]. Apoptosis was confirmed through flow-cytometry analysis after staining with Annexin V and Propidium Iodine.

### Efferocytosis assay and gating strategies

Apoptotic MLE cells were stained with Carboxyfluorescein Succinimidyl ester (CFSE) according to the manufacturer’s recommended protocol (Life technologies, CA). CFSE labeled apoptotic cells were then intranasally delivered (10^6^ cells/mouse) into alveolar space under deep general anesthesia. After 1h, BALs were collected and BAL cells were stained with F4/80 antibody. Fujimori et al gating strategy was used as shown in the S1 Fig[18]. We gated on autofluorescent cells as a very convenient feature for identification of AMs in BALs by flow cytometry and used F4/80 as a de-facto marker for lung phagocytes including macrophages [19]. The CD11c^hi^, CD11b^lo^ cells in the BALs were considered alveolar macrophages whereas non-alveolar macrophages were CD11b^hi^, CD11c^int/lo^. We also looked at Ly6G and CD11b to identify neutrophils. CD11c was also used as a second marker that is also an endocytic receptor that in the lung is expressed by alveolar macrophages [20]. Other markers such as PPARγ were also measured that are exclusively expressed by alveolar macrophages[21].We gated on CFSE+ (apoptotic cells) and F4/80+(AMs) cells. The efferocytosis efficiency was calculated as previously described [17].

### Confocal microscopy

Apoptotic MLE cells were labeled with Annexin V-Biotin (Miltenyi Biotech inc., CA),and then incubated with pHrodo Red Avidin (Invitrogen). The pHrodo stained apoptotic cells were administered intranasally (10^6^ cells/mouse). BAL cells were collected and stained with DAPI and CD11c and subjected to confocal microscopy. Zeiss confocal system (LSM 510 META) with an inverted microscope was used for imaging. The images were processed using LSM Zen 2009 software and compiled using Adobe photoshop software.

### Western blotting

APs were lysed and total cellular protein was isolated using M-PER reagent (Thermo Scientific Inc.). Equal amounts (15μg) of protein from each sample was loaded on 4–15% SDS-polyacrylamide gel (Bio-Rad Inc., CA), separated and transferred onto a PVDF membrane (Bio-Rad Inc., CA). Expressions were detected by incubating the PVDF membrane with specific antibodies to PPAR-α (1:1000), PPAR-δ (1:1000), Tim 4(1:500) (abcam Inc., MA) and PPAR-γ (1:1000) (Cell signaling technologies Inc.). Signals were visualized by chemiluminescent detection.

### In vivo blocking of TIM4

TIM4 blocking antibody or IgG control (200μl,BioXCell, NH) were injected intraperitoneally 1 day after BCG vaccination and 1 day prior to efferocytosis assay. The assay was performed using flow cytometry as described earlier. For IAV survival experiments, mice were treated at days -1, 2, 5 and 8 post IAV infection with either anti-TIM4 blocking antibody or an isotype control. Mice were monitored for 4 weeks for weight loss and mortality.

### Quantification of small-GTPases and cytokine expression

BAL cells were collected from different groups prior to and after IAV infection (2DPI). Total RNA was extracted using Trizol®. cDNA synthesis was carried out using Superscript first strand synthesis kit (Life Technologies, CA). iTaq universal SYBR® Green Supermix (Bio Rad, CA) was used for quantitative RT PCR and specific primers for Rab5, Rab7, TNF and IL-10 genes. β-Actin was used as a house keeping gene.

### RhoA and Rac1 activity

BAL cells were collected from BCG- and PBS-treated mice before and after IAV infection (2DPI). Cell lysates were prepared and Rac1 and RhoA activity was measured using G-LISA RhoA activation assay protocol as described by manufacturer (Cytoskeleton, Co).

### Measurement of cytokine concentrations

BAL fluids were collected at different time points and stored at -80^°^C. Levels of TNFα and IL-10 were measured by enzyme-linked immunoassay (eBiosciences, CA) [15].

### Statistical analysis

GraphPad Prism 4.0was used to perform statistical analysis. Student t-test, One-way and two-way ANOVA were used for analysis. Statistical values ≤0.05 were considered significant.

## Results

### Pulmonary delivery of BCG enhances efferocytosis by APs

**Fig 1:**
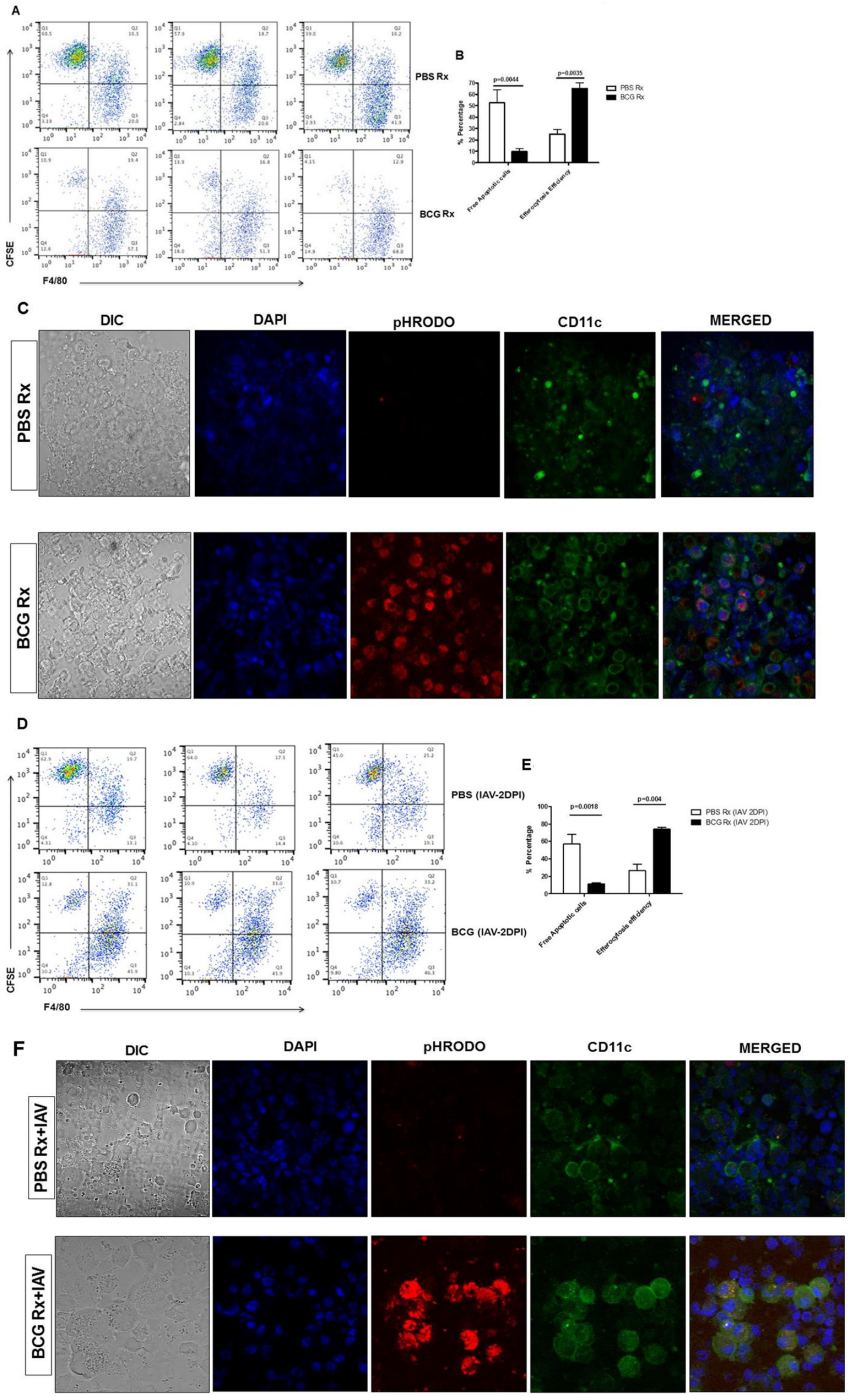
Delivery of BCG to alveolar space enhances efferocytosis by APs. Staurosporine-induced apoptotic mouse lung epithelial (MLE) cells were labeled with CFSE and intranasally transferred to mice treated intranasally with BCG or PBS. Bronchoalveolar lavage (BAL) cells were collected 1 h later and stained with F4/80. (A) Flow cytometry analysis of individual mice is shown. (B) Average of free CFSE+apoptotic MLE cells and efferocytosis efficiency from panel A. Efferocytosis efficiency was calculated as percent of efferocytosed CFSE+ apoptotic cells (CFSE+ and F4/80+F4/80+double-positive cells)/total percent of CSFE^+ve^ cells. A representative of two to four independent experiments with 3–5 mice per group are shown. Error bars show SEM. (C**) Localization of apoptotic cells insideAPs.** WT mice were treated as in panel A. Apoptotic MLE cells were stained with red pHrodo® and administered intranasally to PBS-treated (upper row) control and BCG-treated (lower row). BAL cells were collected and subjected to confocal microscopy.60x Magnification. A representative of two independent experiments with 3–4 mice per group is depicted. (D-F**) Pulmonary treatment with BCG rapidly and efficiently boosts efferocytosis by APs in disease condition.** WT mice were treated intranasally with BCG or PBS. Two days later, all mice were infected with PR8 strain of influenza virus. Two days after IAV infection, all mice received CFSE-labeled apoptotic MLE cells intranasally and the apoptotic cell clearance was measured by flow cytometry, as described above. (D) Flow cytometry analysis of each individual mouse either treated with PBS (top row) or BCG (bottom row) is shown. (E)Average percent of free CFSE+ apoptotic MLE cells and efferocytosis efficiency from panel (D) are shown. Efferocytosis efficiency of APs was calculated using the formula from panel B. A representative of three to four independent experiments shown with 3–5 mice per group. Error bars show SEM. (F**) Visualization of the engulfed apoptotic cells inside the APs in BCG vaccinated and control mice after IAV infection.** WT mice were treated as in panel D. Two days after IAV infection, all mice received pHrodo®-labeled apoptotic MLE cells intranasally and the fate of apoptotic cells inside the AMs of PBS-treated (upper row) control and BCG-treated (lower row) mice was studied using confocal microscopy, as described for panel1C. 60x magnification. A representative of two independent experiments with 3 mice per group is depicted.

Bacillus Calmette-Guerin (BCG) has been shown to increase the recruitment of macrophages as well as macrophage activation [14]. To investigate whether BCG can enhance efferocytic ability of alveolar phagocytes, WT mice were treated with BCG intranasally (IN) as described in materials and methods. Efferocytosis activity of APs was evaluated 2 days later. Apoptotic MLE cells were then labeled with CFSE and transferred into the alveolar space of BCG-immunized and their PBS-treated counterparts. One hour after the cell transfer, bronchoalveolarlavage (BAL) cells were collected, stained for F4/80 and subjected to flow cytometry. Antibody-based recognition (flow cytometry and microscopy) of cell-surface markers is widely used for characterization/identification of immune cells, however, overlap in the expression of markers by different cell types and have resulted in a lack of clearly defined signatures for myeloid cell subsets particularly in the lung. We used F4/80 as a de-facto marker for lung phagocytes including macrophages in flow cytometry[19]. CD11c was used in confocal microscopy as a second marker that is widely expressed on mice macrophages, granulocytes, and DCs and is also an endocytic receptor that in the lung is expressed by alveolar macrophages [20]. Gating strategies have been delineated in S1 Fig. Delivery of BCG into the alveolar space significantly increased the uptake and clearance of apoptotic MLE cells by APs. BCG-immunized mice showed significantly superior efferocytosis as un-ingested apoptotic cells in BALs of BCG–immunized mice radically reduced compared with PBS-treated mice [9.65±2.88 vs. 52.4.0±6.7, respectively; Fig 1A and 1B (upper left quadrants)]. This indicated increased uptake of apoptotic cells in BCG-immunized group. The efferocytosis efficiency of APs in BCG-immunized mice was significantly higher compared with that of PBS-treated mice (65.17±4.79 vs. 25±2.35) (Fig 1B).

### Tracking ingested apoptotic cells in the APs

In order to confirm that apoptotic cells were ingested byAPs, we stained apoptotic MLE cells with red pHrodo® dye before delivery to alveolar space. pHrodo® dyes are non-fluorescent at neutral pH and fluoresce brightly in acidic environment inside the phagocytes[17]. Apoptotic MLE cells stained with red pHrodo® were delivered IN to BCG-vaccinated and PBS-treated mice. BAL cells were collected, stained for CD11c and DAPI and subjected to confocal microscopy. Expression of CD11c as a marker for APin BCG-vaccinated mice significantly increased compared to those of PBS-treated control mice (Fig 1C and S2 Fig). pHrodo®-stained apoptotic MLE cells were fluorescing only in the APs from BCG-vaccinated mice (Fig 1C). Profoundly increased level of red pHrodo fluorescence in APs (Fig 1C, lower panel) was indicative of presence of large amounts of red pHrodo®-labeled apoptotic cells in a low pH environment inside the cell (Fig 1C, lower panel). In all cases, pHrodo red fluorescence co-localized with CD11c marker (Fig 1C, lower panel), depicting that the cell with intracellular pHrodo®-stained apoptotic cells were APs.

### Role of BCG-induced efferocytosis by APs in protecting lung

We and others have shown pivotal roles of APs in protecting lung against lethal influenza and bacterial pneumonia [15–17,22]. Hence, we next decided to assess the efferocytosis efficacy of APs in BCG- and PBS-treated control mice in a disease condition such as lethal IAV infection. Alveolar epithelial cells are the main targets for IAV and damages to lung epithelial barrier are the major cause of respiratory failure during IAV infection [23]. WT mice were treated with PBS or BCG intranasally and 48 hours later were infected with 2LD50 of the mouse adapted PR8 strain of IAV. Forty eight hours after IAV infection, we overwhelmed the AMs by delivering CFSE-labeled apoptotic MLE cells into the alveolar space. Their BAL cells were collected 1 hour after the cell transfer and stained for F4/80 surface marker. BCG vaccinated IAV-infected mice had significantly less apoptotic MLE cells (CFSE+) in their BALs compared to PBS-treated IAV-infected control group [11.3%±0.53 vs. 57.3%±6.15, Fig 1D (upper left quadrants) and 1E]. Moreover, efferocytosis efficiency by APs in BCG vaccinated IAV-infected mice was significantly better than that of the APs from their PBS-treated IAV-infected counterparts (74.07%±1.29 vs. 26.7%±4.19, Fig 1E). This showed that delivery of BCG to alveolar space enhanced efferocytic activity of APs and resulted in effective uptake of apoptotic lung epithelial cells after IAV infection even in a condition in which the lung was inundated by both endogenous and exogenous apoptotic epithelial cells.

We also assessed whether the apoptotic cells were inside the APs after IAV infection by staining apoptotic MLE cells with red pHrodo dye before delivery to alveolar space as described for Fig 1F. Mice were infected with lethal PR8 (2 LD50) 2 days after intranasal treatment with either BCG or PBS. pHrodo®-stained apoptotic MLE cells were transferred into their alveolar space 48 hrs after IAV infection. BAL cells were collected, stained with CD11c and subjected to confocal microscopy. Expression of CD11c and the size of CD11c+ cells were significantly increased in BCG-immunized IAV-infected mice compared to those of PBS-treated IAV-infected control mice (Fig 1F). Also, pHrodo®-stained apoptotic MLE cells were fluorescing brightly in the APs from BCG-treated IAV-infected mice (Fig 1F, lower panel). Extremely bright red fluorescence in APs was indicative of presence of large amounts of red pHrodo®-labeled apoptotic cells in a low pH intracellular environment. Again, in all cases, pHrodo® red fluorescence co-localized with CD11c marker (Fig 1F, lower panel), indicating that the intracellular apoptotic pHrodo®-stained cells were internalized by APs.

### Delivery of BCG to the lungs protects WT mice against IAV pneumonia

**Fig 2:**
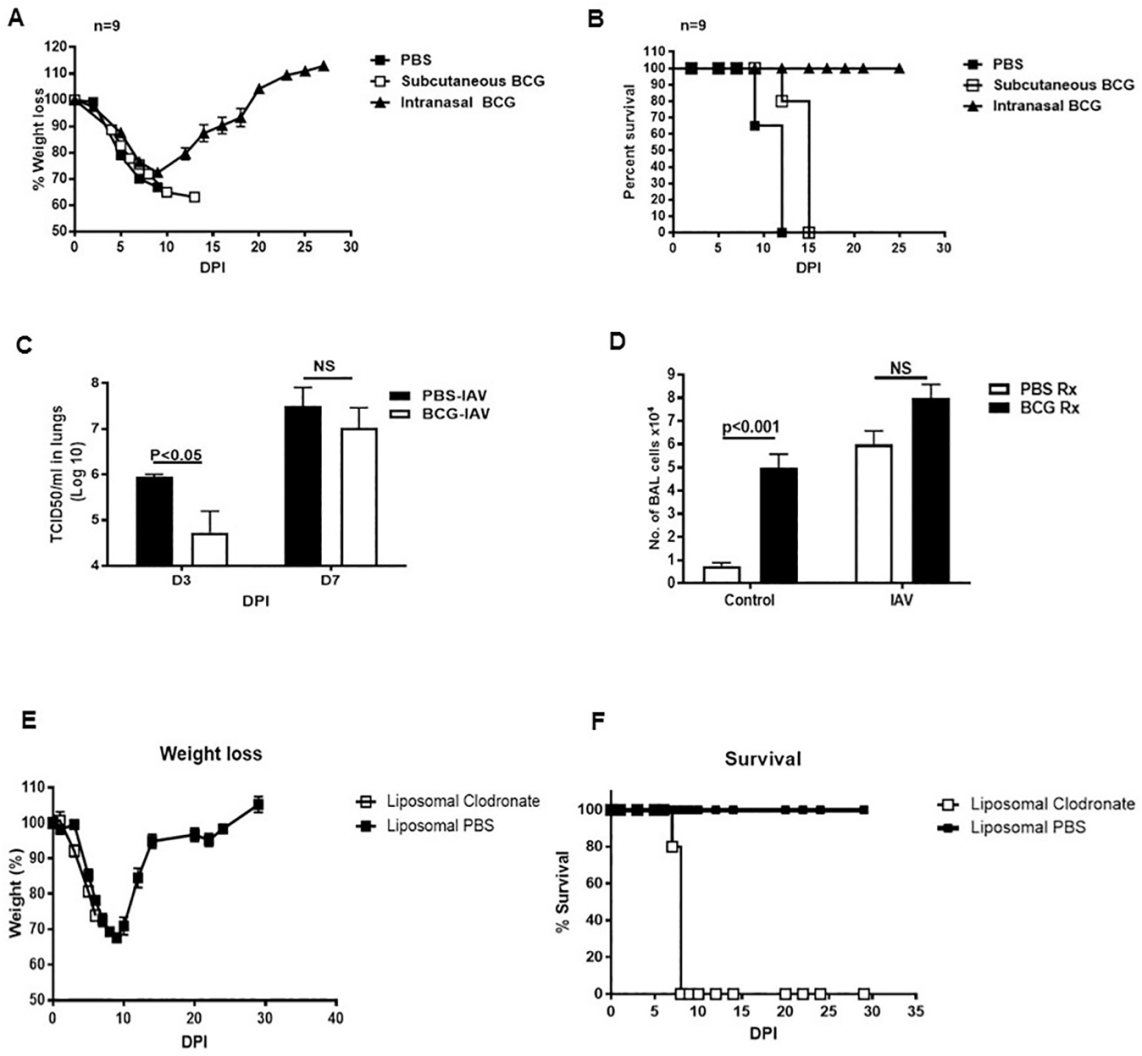
Intranasal treatment with BCG protects mice against lethal influenza infection. WT mice (9 per group) were treated with either PBS or BCG (either intranasally or subcutaneously), and all mice were infected with a lethal dose (2 LD50) of influenza PR8 virus. Weight loss (A) and mortality (B) were recorded daily. (C)Different groups of mice were treated as for panels A and B. Three and 7 days after influenza infection, mice were sacrificed, lung homogenates were generated and virus load was measured. Depicted data are average of 5 mice per group and error bars show SEM. NS = Not significant. (D) BAL cells from a group of BCG- and PBS-treated mice prior to and after IAV infection were quantified. Depicted data are mean of 5 mice per group. Error bars show SEM. (E-F) **Depletion of APs abrogated BCG-mediated protection against lethal influenza infection.** Mice were intranasally treated with BCG and 24 hrs prior to influenza PR8 infection (2LD50) they were treated with either liposomal clodronate to deplete APs or PBS-liposome as control. Infected mice were monitored for (E) weight loss and (F) mortality. Mean of weight loss with SEMs are depicted. n = 5. DPI: Days Post Infection.

To assess the role of efferocytosis by AP in protecting hosts against an acute pneumonia, we treated WT mice with either BCG (intranasally and subcutaneously) or PBS (intranasally). Two days later, all mice were infected with lethal dose of IAV (2 LD50). Infected mice were monitored daily for morbidity and clinical signs of influenza pneumonia. All infected mice lost weight, indicating 100% infection efficiency (Fig 2A). All PBS-treated IAV-infected mice succumb by day 10 post IAV-infection. However, 100% of mice immunized intranasally with BCG survived lethal IAV infection (Fig 2B). All mice immunized intranasally with BCG showed significant weight loss, however by day 9 post-infection, they started to gain their weight back and by day 21 post IAV infection they started to pass their pre-infection weight (Fig 2A). We also tested the notion whether subcutaneous immunization with BCG can provide the same protection as intranasal delivery. Subcutaneous immunization did not provide any protection against IAV and 100% of mice immunized subcutaneously with BCG died 15 days after IAV infection (Fig 2A and 2B).

We also measured the viral load in the lung homogenates of intranasal BCG- and PBS-treated IAV infected mice 3 and 7 days post IAV infection. The loads of influenza virus was lower in BCG immunized group compared to PBS-treated group, however, the viral loads differences were only statistically significant at 3 days post IAV infection (Fig 2C). Intranasal BCG immunization significantly increased number of BAL cells compared to PBS-treated group (Fig 2D). However, the number of BAL cells after IAV infection was comparable between both BCG- and PBS-treated groups (Fig 2D).

To confirm the role of APs in protecting BCG-vaccinated mice against IAV, we depleted APs from mice vaccinated intranasally with BCG one day before IAV infection by liposome clodronate [15,16]. Control mice were treated with Liposome PBS. All BCG-vaccinated mice treated with liposome clodronate died 8 days after lethal IAV infection whereas 100% of BCG-vaccinated liposome PBS-treated mice survived (Fig 2E and 2F).

### Effect of BCG on receptors for apoptotic cell on APs

**Fig 3:**
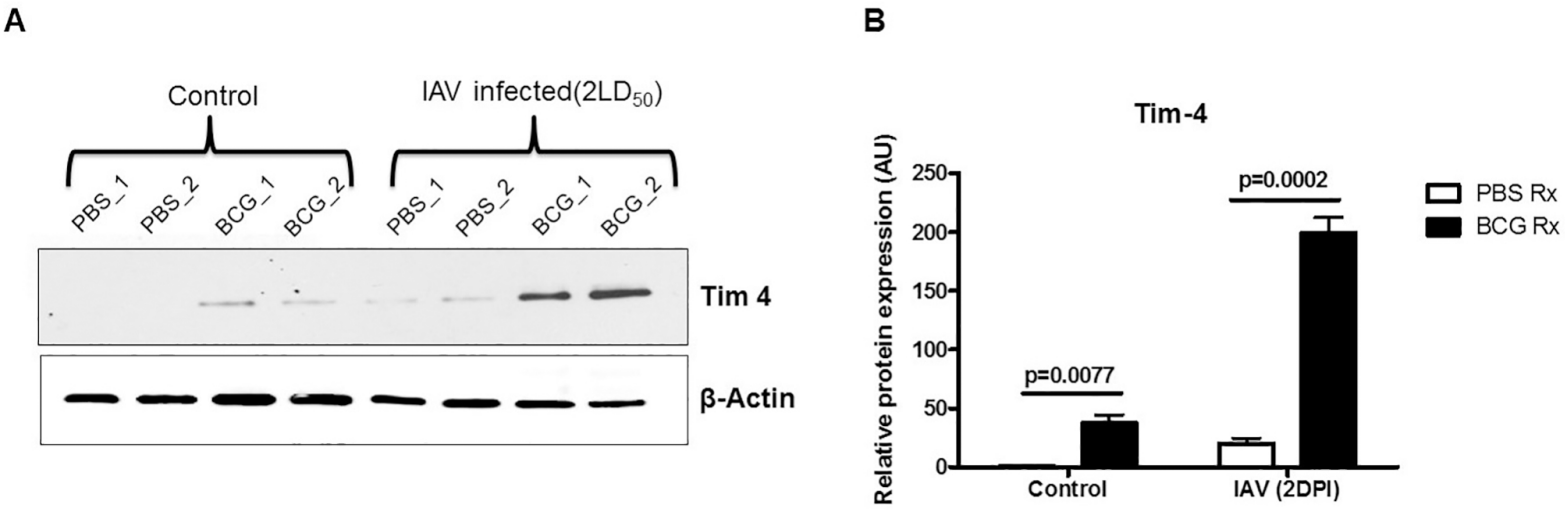
BCG vaccination increases Tim4 expression in APs. **(**A) Expression of Tim-4 was determined in BCG vaccinated and control PBS treated mice before and after influenza infection using western blotting. WT mice were treated with either BCG or PBS and infected with lethal PR8 as described in the legend of Figs 1D and 2. Two days after influenza infection, BAL cells were collected, pooled and lysed as delineated in materials and methods. Each lane is pool of BALs from 5 mice. Equal amount of protein extracts from each group were loaded into each lane. (B) Densitometry analysis of band-intensity using Image Lab software (Molecular Imager Gel Doc system from Bio-Rad) is shown. Data is representation of band-intensities of 3 independent blots. Error bars show SEM. DPI: Days Post Infection.

APs provide the first line of host defense by internalizing and degrading different pathogens, apoptotic cells, and inhaled particles. Different surface receptors are involved in phagocytosis and efferocytosis by APs [24,25]. We examined the expression of surface molecules on APs that bind to apoptotic cells and initiate their uptake [17]. APs from BCG-vaccinated mice and their PBS-treated counterparts were stained for expression of CD51, CD36, CD16/32 and TIM4. BCG-vaccination only increased the expression of TIM4 (Fig 3) and expressions of CD51, CD36 and CD16/32 were similar on APs from BCG-vaccinated and PBS-treated groups (S3 Fig). BCG-treatment significantly increased TIM4 expression by APs compared to APs from PBS-treated mice (Fig 3). Expression of TIM4 was slightly increased in the APs from PBS-treated mice2 days after IAV-infection but it was much more pronounced in APs of BCG-immunized IAV-infected mice (Fig 3).

### Neutralizing TIM4 reduced efferocytosis by APs

**Fig 4:**
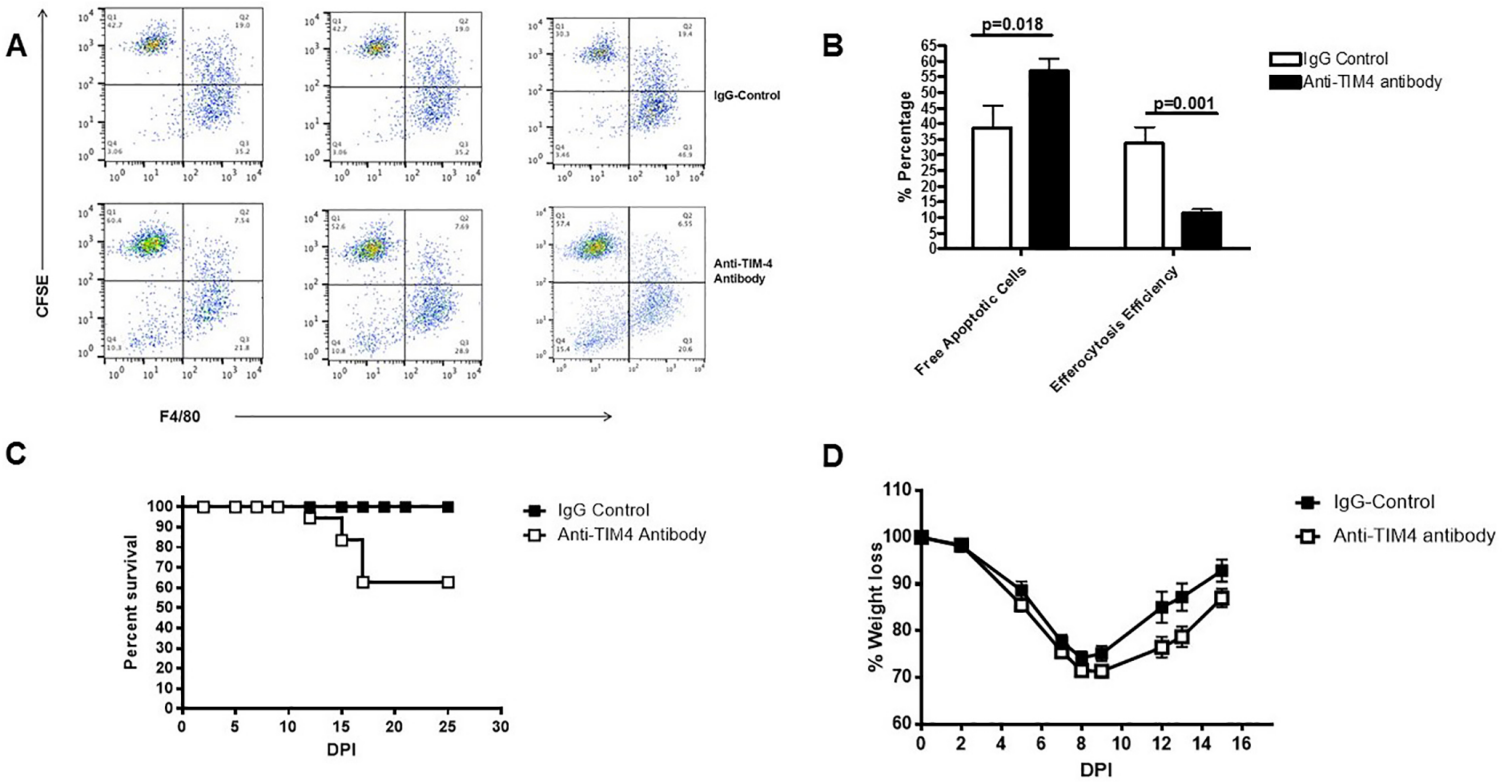
Neutralizing Tim4 reduces BCG mediated efferocytosis by APs. WT micewere treated intranasally with BCG and 24 hours later were treated with 200μg of either anti-TIM4 blocking antibody or isotype control. Next day, all mice were infected with lethal dose of PR8 influenza virus (2LD50). Antibody treatment continued with 3 days intervals at days 2,5,and 7 post PR8infection. (A) Apoptotic MLE cells labeled with CFSE were intranasally transferred to isotype (top row) or anti-Tim4 (bottom row) treated mice, BALs were collected an hour later andefferocytosis efficiency of AMs was evaluated using flow cytometry, as described for Figs 1 and 3. (B) Average percent of free CFSE+ apoptotic MLE cells and efferocytosis efficiency from panel (A) are shown. Efferocytosis efficiency of AMs was calculated as described for Fig 1C and 1F. A representative of two independent experiments is shown with 3 mice per group. Error bars show SEM. (C and D)Weight loss and mortality were recorded daily. A representative of two independent experiments with similar results is shown. n = 10.

To confirm the role of TIM4 in efferocytosis by APs, we used neutralizing strategy. BCG-immunized mice were treated with anti-TIM4 antibody or IgG-isotype control 24 hrs after BCG vaccination and CSFE-labeled apoptotic MLE cells were given IN as described for Fig 1. Neutralizing TIM4 significantly reduced the efferocytosis of apoptotic MLE cells by APs in BCG-immunized group (Fig 4A and 4B). BAL cells from BCG-immunized mice treated with TIM4-neutralizing antibody had significantly more free-apoptotic cells than their isotype-treated counterparts (Fig 4A and 4B). Also, efferocytosis efficiency of APs from BCG-immunized mice treated with TIM4-neutralizing antibody was significantly lower than that of APs from BCG-immunized mice treated with IgG-isotype antibody (Fig 4B).

We next investigated the role of TIM4 in protecting the host against lethal IAV infection. Treating BCG-immunized mice with anti-TIM4 antibody reduced the protection of intranasal BCG-immunization from 100% to 60% (Fig 4C). Moreover, BCG-immunized IAV-infected mice that were treated with anti-TIM4 antibody showed more severe clinical signs compared to their isotype-treated counterparts as indicated in their weight loss and recovery. Anti-TIM4 treated BCG-immunized group lost more weight by day 9 post IAV-infection and started to regain their lost weight much slower than the isotype antibody-treated group (Fig 4D).

### BCG vaccination affects expression of GTPases by APs

**Fig 5:**
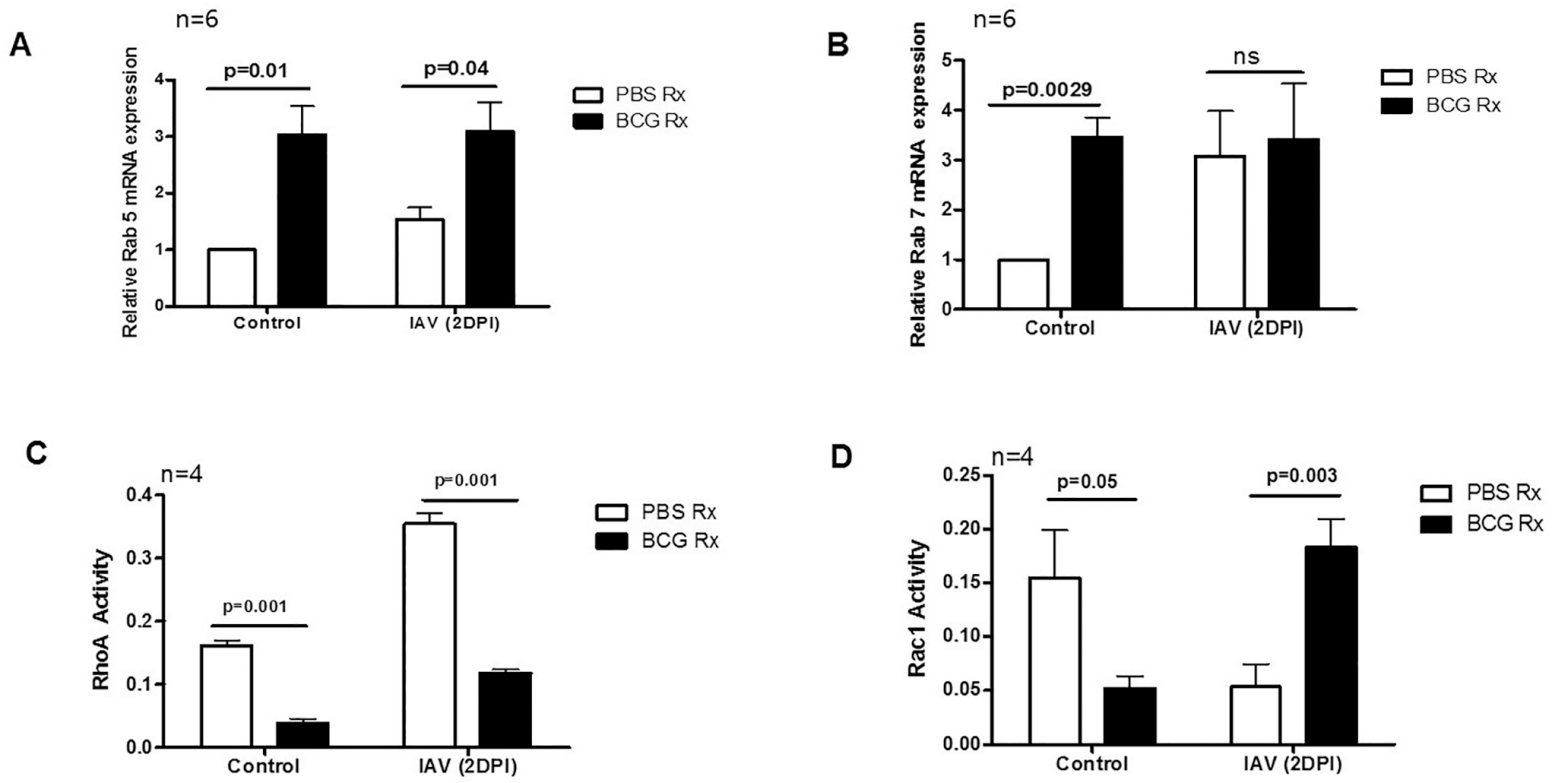
Effects of BCG on expression of small GTPases in APs. APs from WT mice treated with either PBS or BCG were collected prior to and 2 days after influenza infection. Total RNA was extracted, cDNA was synthesized, and quantitative reverse transcription-PCR was carried out. Beta Actin was used as a housekeeping gene and expression levels of (A) Rab5 and (B) Rab7 were normalized against the Beta Actin mRNA level. (C) Rho A and (D) Rac1activity of AMs from BCG- or PBS-treated mice prior to and 2 days after influenza infection was measured using G-LISA RhoA and Rac 1 activation assay protocol, respectively. A representative of two independent experiments with 4–6 mice per group are shown with error bars as SEM. DPI: Days Post Infection.

Endomembrane trafficking in eukaryotic cells are being regulated by small GTPases such as Arf, Rab, Ras, Ran, and Rho. In eukaryotic cells, Rab GTPases play important roles in phagocytic trafficking by mediating vesicle formation, maturation, and transport [26,27]. It is known that in the phagocytosis/efferocytosis pathways for recycling purposes, cargo is transported from Rab5 to Rab4 and Rab11 domains, but for degrading purposes cargo moves from Rab5 to Rab7 domains. Hence, Rab7 has a key regulating role in phagolysosome fusion transport of lysosome-destined enzymes and internalized surface proteins to the lysosome through the endocytic pathway [27,28]. We measured mRNA expression levels of Rab5 and Rab7 in the APs of mice either treated with BCG or with PBS, prior to and 2 day after IAV infection. BCG immunization significantly increased the expression of Rab5 and Rab7 in APs, however, 2 days after IAV infection the mRNA levels of Rab7 in APs from BCG- and PBS-treated mice were not significantly different (Fig 5A and 5B). When we assessed the activity of Rac1 and RhoA, APs of BCG-immunized mice had increased Rac1 activity after IAV infection however the RhoA activity was significantly higher in PBS-treated groups compared to BCG-treated ones (Fig 5C and 5D).

### BCG reduces inflammation in the lungs

**Fig 6:**
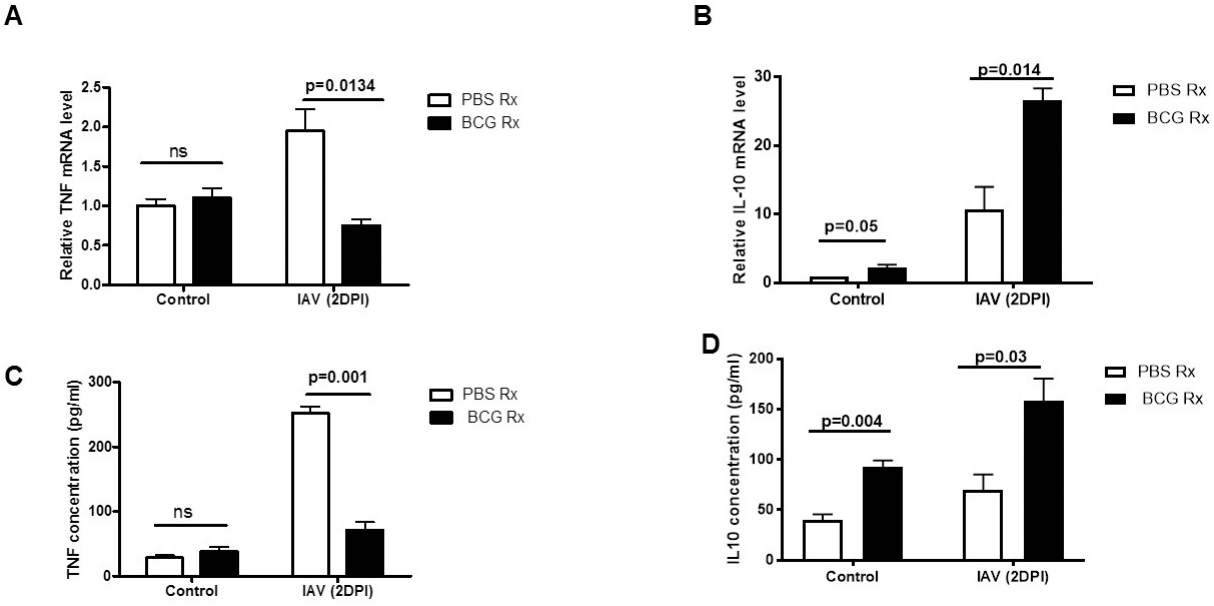
Effects of BCG on TNF and IL10 cytokines in the lungs. WT mice were treated as in Fig5. mRNA expression of TNF (A) and IL10 (B) in the BAL cells and protein levels in the BAL supernatants (C and D) were measured by real time PCR and ELISA, respectively. Mean of 6 mice/group is shown with error bars showing SEM. DPI: Days Post Infection.

Efferocytosis is known to be essential in reducing inflammation and facilitating tissue homeostasis and repair. TNF inhibits clearance of apoptotic cell in the lung and exacerbates acute inflammation [29]. We measured mRNA and protein levels of TNF and IL10 in the BAL cells and supernatants, respectively from BCG- and PBS-treated mice prior to and after IAV infections. TNF mRNA and protein levels from BALs of BCG-immunized mice were significantly lower than that from BALs of PBS-treated control group (Fig 6A and 6C). Moreover, the levels of IL10 were significantly higher in the BCG-immunized group compared to PBS-treated control mice (Fig 6B and 6D).

### Overexpression of nuclear receptors in APs of BCG-vaccinated mice

**Fig 7:**
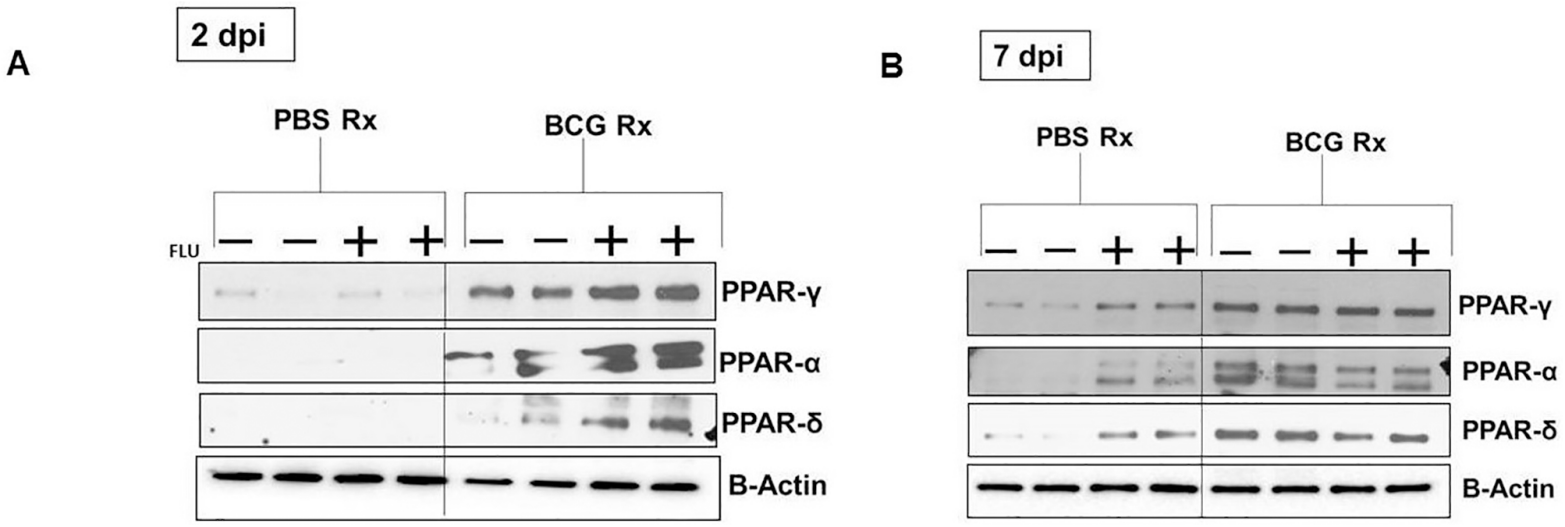
Expression of PPAR nuclear receptor in BAL samples of BCG- and PBS-treated mice. Expressions of PPAR- α/β/δ in mouse alveolar phagocytes were measured using western blotting. Each lane is pool of BALs from either 3 BCG- or 3 PBS-treated mice that were collected prior to and 2 (A) and 7 (B) days after influenza PR8 infection. Cell lysates were subjected to western blot analyses. β-actin was used as control. Representative of 2 independent experiments with similar results is shown. DPI: Days Post Infection.

Nuclear receptors are a superfamily of ligand-activated transcription factors that their roles in inflammation and tissue homeostasis during efferocytosis have recently been emerged [30]. Hence we evaluated the expression of peroxisome proliferator-activated receptors (PPARs) in APs from BCG- and PBS-treated control mice. APs were collected 48hrs after BCG- or PBS-treatment and PPAR levels were measured by western blotting prior to and after IAV infection. BCG-treatment significantly increased the expression of PPARα, PPARδ and PPARγ in APs compared to their PBS-treated counterparts prior to and 2 and 7 days after IAV infection (Fig 7A and 7B). Expression of all three PPAR-α, -δ, and -γ were also increased 7 days post IAV infection in PBS-treated group but in lesser extent than in their BCG-treated counterparts (Fig 7B).

## Discussion

Published data from our lab and others have shown the essential role of APs in lung innate immunity and in protecting host against lethal pneumonias [15–17,22,31]. APs contribute to the lung innate immunity and maintaining homeostasis by several mechanisms. APs play an important role in reducing inflammation in the lungs by ingestion of apoptotic cells, termed “efferocytosis” [5,17].

In the current report, we continued our studies on the innate immunity of the lungs and assessed the effects of BCG on APs. Our data demonstrated that intranasal immunization with BCG increased efferocytosis by APs and conferred protection against lethal IAV pneumonia (Figs 1 and 2). Mice immunized with BCG and 2 days later were challenged with lethal IAV (2 LD50) illustrated 100% protection against lethal influenza infection whereas 100% of control group succumb (Fig 2A and 2B). This robust protection only 2 days after intranasal delivery of BCG strongly suggested that the BCG-boosted innate immunity in the lungs was responsible for the protection of host against lethal IAV infection.

Influenza infections disrupt lung homeostasis by targeting host lung epithelial cells. Influenza-infected lung epithelial cells then become apoptotic and necrotic which trigger excessive inflammation and cytokine storms in the lung [32–34]. Maintaining lung homeostasis is critical to normal respiratory function, particularly during crisis such as IAV pneumonia. Alveolar macrophages (AM) are the main phagocytes in the alveolar space and uptake apoptotic and necrotic cells through a process known as efferocytosis to maintain and/or restore lung homeostasis [32,33]. Efferocytosis by alveolar macrophages reduces oxidant damage, initiates anti-protease activity, and stimulates vascular endothelial growth factor[35,36]. Increased efferocytosis by AMs results in protection against lethal acute lung injury due to pulmonary infections [16,17]. Also, defective efferocytosis by AMs results in chronic lung inflammation and significantly increases the likelihood of developing chronic obstructive pulmonary disease (COPD), acute lung injury and cancer [1–4]. It is therefore critical to develop different interventions to boost efferocytosis by APs to prevent and treat these conditions.

BCG has been used for many years as an anti-tuberculosis vaccine and as an immunotherapeutic agent to treat melanoma cases, and treat and prevent the recurrence of superficial bladder tumors [8–11]. Protective role of BCG against influenza infections in mice and unintended beneficial effects of BCG vaccinations in humans have been demonstrated by others [12,13]. BCG’s non-specific protections against other diseases are mostly through enhancement of macrophage recruitment and function in the site of infection [14]. In our novel approach, we tested the role of BCG on efferocytosis by APs and mechanisms by which it protects against lethal influenza pneumonia. We tested the role of efferocytosis by APs in BCG-immunized mice and in all cases, APs from BCG-immunized mice showed significantly more efficient efferocytic activity compared to the APs from their PBS-treated counterparts (Fig 1).

We also studied the importance of AP efferocytosis in a disease condition and evaluated the efferocytosis activity of APs in BCG-immunized and PBS-treated control mice after lethal IAV infection. First, depletion of APs totally abrogated BCG-induced protection against lethal IAV infection (Fig 2E and 2F). We also delivered CFSE-labeled apoptotic MLE cells into alveolar space of BCG and PBS treated mice two days after lethal IAV infection. Considering that IAV infection causes apoptosis of the hosts’ lung and airway epithelial cells [37], adding CFSE+ apoptotic MLE cells into alveolar space overloaded the APs of recipient mice. Nonetheless, APs from IAV-infected BCG-immunized mice removed free CFSE+ apoptotic MLE cells significantly faster than APs from IAV-infected PBS-treated group. BCG-immunized IAV-infected mice had also more efferocytic APs (double positive CFSE+ and F4/80+ cells) than IAV-infected PBS-treated control groups and superior efferocytosis efficiency than control group (Fig 1D–1F). This clearly demonstrated that even in a lethal disease condition such as IAV infection in which APs were overwhelmed by exogenous apoptotic cells (CSFE-labeled MLE cells) and endogenous IAV-infected apoptotic lung and airway epithelial cells, the APs from mice that were treated intranasally with BCG for only 2 days prior to IAV-infection were able to uptake and clear apoptotic cells and maintain lung homeostasis.

To prove that apoptotic cells had been internalized by APs and evaluate the efferocytosis efficiency of each individual cell, we used red pHrodo® dye to stain apoptotic MLE cells and used confocal microscopy. pHrodo® dyes are non-fluorescent at neutral pH such as cell culture medium but fluoresce intensely in acidic milieu such as phagolysosome. APs from BCG treated mice had markedly larger number of red pHrodo® fluorescing cells compared to APs from PBS-treated group both prior to and after IAV infection (Fig 1C and 1F). All pHrodo® red fluorescing cells were co-localized with CD11c and DAPI (the nuclear staining dye) illustrating that pHrodo®-stained apoptotic MLE cells were inside APs and in a low pH environment.

Efficient recognition and clearance of the cells such as aged and apoptotic cells are important tasks of the immune system for maintaining the homeostasis of an organ. Extremely rapid apoptosis of lung epithelial cells in influenza and other lung injuries can trigger an unrestrained inflammatory response and cytokine storm [7,38]. Also, if apoptotic cells are not rapidly and efficiently removed, there is a risk of progression to secondary necrosis leading to the loss of integrity of cell membranes and release of cytoplasmic and nuclear components that serve as ligands for pro-inflammatory receptors [39,40]. Defective efferocytosis in smokers and patients with airway diseases has been shown in several studies [39–41]. For instance, cigarette smoke decreases alveolar macrophages clearance of apoptotic cells and COPD patients who are active smokers and makes them prone to severe exacerbations. Acute phase-reactant alpha-1 antitrypsin (A1AT) reduces the severity of COPD exacerbations in A1AT deficient (AATD) individuals. Recently, Serban et al have shown that A1AT significantly improves efferocytosis of AM from active smokers particularly when delivered through inhalation [42]. During acute infections such as influenza that generate large number of apoptotic cells in a short period of time, there is an imperative need for the clearance of the huge number of apoptotic cells that are generated in the first few days of the infection [37,43,44]. We here report that delivery of BCG into the alveolar space boosted the capacity of AMs to remove apoptotic cells and increased efferocytosis by AMs prevented and/or mitigated acute lung injury caused by lethal IAV infection.

Our results support the notion that clearing apoptotic/necrotic cells and promoting lung homeostasis is a valuable strategy in protecting host against acute and cytopathic intracellular pathogens such as IAV [15–17]. This strategy facilitates maintaining lung homeostasis, reduces dissemination of pathogen and perhaps provides adaptive immunity with sufficient time to clear the pathogen. We measured viral burden of influenza A PR8 in the lung homogenates of both BCG-immunized and PBS treated mice after IAV infection. Three days post IAV infection viral loads were significantly higher in PBS-treated mice compared to BCG-immunized group. Containing the infection is likely due to increased efferocytosis of IAV-infected apoptotic epithelial cells by APs in BCG-immunized mice that reduced the viral burden in the lungs and prevented the spread of IAV into the uninfected epithelial cells [43,44].

Several membrane-associated receptors on AMs [such as CD36, CD51/CD61, CF transmembrane conductance regulator, class A scavenger receptors, CD31, lung collectin associated receptor, CD91 and CD44]are involved in recognition of apoptotic cells by AMs and defective efferocytosis in patients with airway diseases has been shown in several studies [33,36,40]. Healthy smokers and COPD patients exhibit reduced CD31, CD91, CD44, and CD71 compared with never-smoker control subjects [40]. In the current report, we have shown that delivery of BCG into the alveolar space increases expression of TIM4 receptor on AMs. We have also shown that treating BCG-immunized mice with anti-TIM4 antibody significantly reduced efferocytosis of apoptotic cells by APs (Fig 4A and 4B).To our knowledge, this is the first report about the role for TIM-4 in efferocytosis by APs and the mechanisms through which BCG increases efferocytosis by APs. Others have shown that Mycobacterial infection increases expression ofTIM-4 and efferocytosis on bystander peritoneal macrophages [32]. Our results are in line with Martin et al findings about increased efferocytosis in peritoneal macrophages and present novel therapeutic opportunities for the treatment of airways diseases due to impaired efferocytosis. However, neutralizing TIM-4 increased the mortality but did not abrogate the protection of BCG-treated group against lethal IAV infection (Fig 4C). This is likely due to redundancy in phosphatidylserine (PS) receptors on APs and that APs do not rely exclusively on TIM-4 as PS receptor, and hence, blocking TIM-4 only partially reduced the BCG-induced protection [44–46]. Moreover, it hints also on the possibilities that BCG may also elevate expression of other PS receptors other than TIM-4 on APs that are unaffected by blocking TIM-4.

Macrophages commonly ingest multiple apoptotic cells [31,47]. Accumulation of apoptotic cells can be noxious for the phagocytes. In order to maintain the homeostasis, phagocytes need to increase digestion and removal of extra metabolites provided by the ingested apoptotic cells that is mediated by PS recognition and nuclear receptors [25]. Nuclear receptors are a superfamily of ligand-activated transcription factors that their roles in inflammation and tissue homeostasis during efferocytosis have recently been emerged [30,31]. There are 58 nuclear receptors, 320 co-regulators and 261 ligands [48]. PPARs have been linked to phagocytosis and polarization of macrophages. Among the nuclear receptors, Glucocorticoid receptors, liver X receptors (LXRs) and PPARs have been linked to the phagocytic capacity and phenotypic polarization of macrophages *in* vitro [49,50]. PPARs have three isoforms (PPARα, PPARδ, and PPARγ), and their endogenous ligands are lipids. We showed that APs from BCG-treated mice had significantly higher levels of PPAR-α, -δ and -γ compared to AMs from their PBS-treated counterparts prior to or after IAV infection (Fig 7). Studies on roles of PPAR-γ on differentiation of fetal monocytes into alveolar macrophages and the critical role of PPAR-δ in the clearance of apoptotic cells have been shown by others [21,31]. However, PPARs have also remarkable immune regulating properties. For instance, in addition to their roles in the metabolism of fatty acids, PPARα regulates *Cpt1*, a gene involved in T cell function and PPARδ represses inflammatory genes through the repressor BCL-6 and orchestrates efferocytosis [31]. PPARγ also has been implicated in macrophage-mediated apoptotic cell clearance and in the function of its target genes, such as upregulating scavenger receptor CD36 [49,51]. However, it is not clear to us whether BCG affected these nuclear receptors and increased efferocytosis or increased effferocytosis by BCG increased the uptake of apoptotic cells and hence increased PPARs was secondary to increased efferocytosis for augmenting digestion and removal of extra metabolites provided by the ingested apoptotic cells. Other studies are underway to answer this question that is outside the scope of the current report.

Our findings demonstrate that harnessing lung innate immunity not only increases lung homeostasis, but also protects the host against lethal diseases such as influenza. Delivery of BCG into alveolar space to boost efferocytosis by APs is in line with other efforts to identify new regimens to boost local innate immunity in the lung and airways to maintain lung homeostasis that is compromised in many airway and lung diseases [17].

Taken together, this report provides new strategy and mechanisms to boost efferocytosis of AMs, and will pave the path for new inventions to maintain lung homeostasis that is compromised in many airway and lung diseases. This report introduces very novel intervention to treat impaired and/or boost efferocytosis of AM that leads to efficient homeostasis of the lung and treatment of lung injury. This will also be a steppingstone to identify other efferocytic-agonist compounds.

## Supporting information

S1 Fig Gating strategy for flow cytometry analysis. (A) Apoptotic MLE cells labeled with CFSE were stained for F4/80 as negative control. (B) Single cell staining of BAL cells for F4/80 prior to and after IAV infection. (C)BAL cells were further analyzed for their surface expression of CD11b and CD11c.
(JPG)

S2 Fig Kinetics of uptake of apoptotic cells by APs. WT mice were treated as in Fig 1D. Apoptotic MLE cells were stained with red pHrodo® and administered intranasally to PBS-treated (upper row) control and BCG-treated (lower row). BAL cells were collected in different time points and subjected to confocal microscopy. 60x Magnification. Pool of BALs from 3–4 mice per group is depicted.
(TIF)

S3 Fig Effect of BCG-treatment of expression of phagocytic receptors on alveolar macrophages. Expression of CD51, CD36, and CD16/32 on AMs of BCG- and PBS-treated control mice were analyzed by flow cytometry. Representative histograms of 3 individual mice for each group gated for F4/80 and respective markers are shown. Isotype antibodies showed baseline staining and were excluded for clarifying the effects of BCG.
(TIF)
