# Supplementary figures and images for: Boosting efferocytosis in alveolar space using BCG vaccine to protect host against influenza pneumonia
Source: PLoS One. 2017 Jul 7;12(7):e0180143. doi: 10.1371/journal.pone.0180143 (PMC5501455; doi:10.1371/journal.pone.0180143)

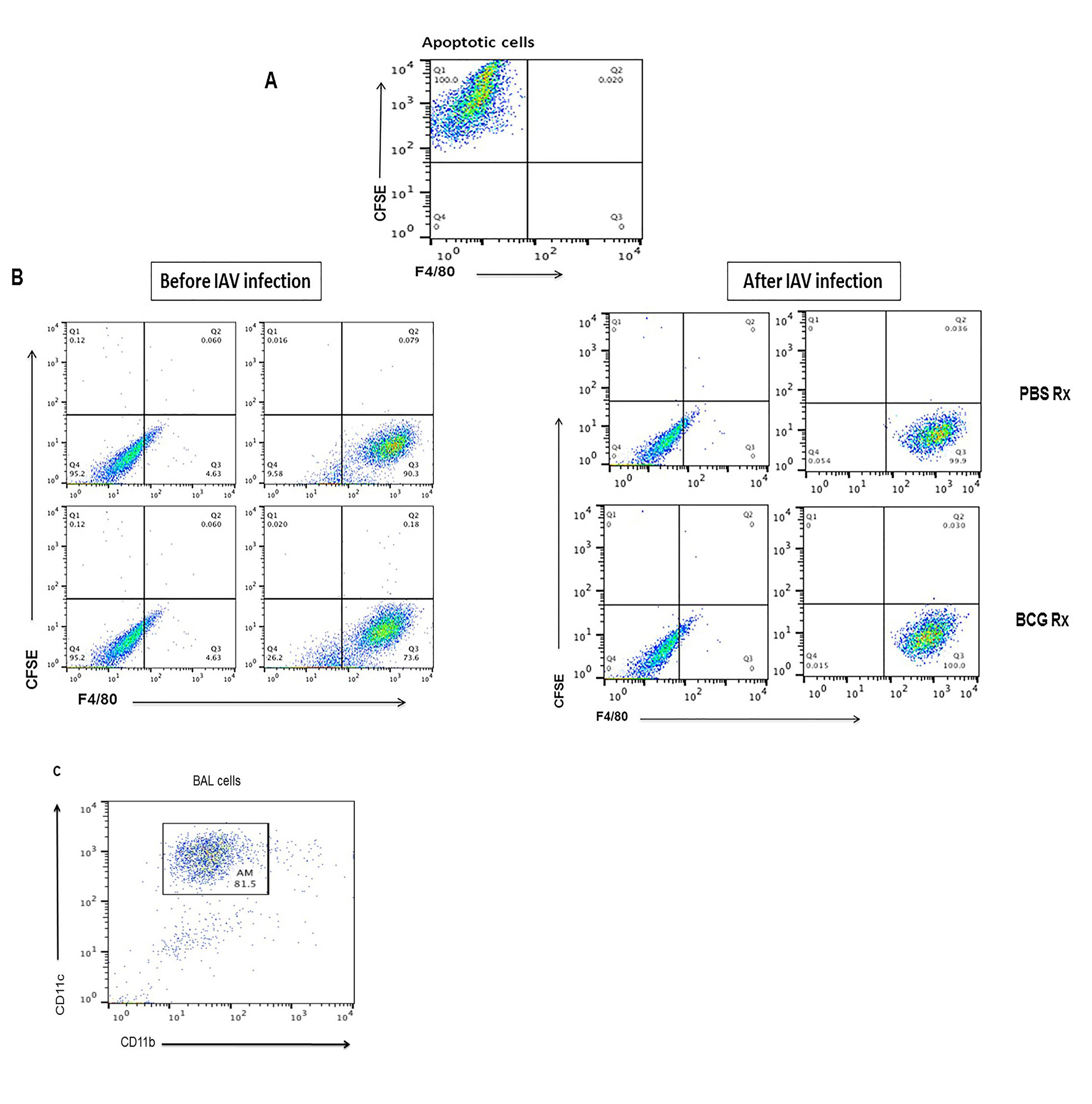

Supplement: S1 Fig — (A) Apoptotic MLE cells labeled with CFSE were stained for F4/80 as negative control. (B) Single cell staining of BAL cells for F4/80 prior to and after IAV infection. (C)BAL cells were further analyzed for their surface expression of CD11b and CD11c. (JPG) [file pone.0180143.s001.jpg]

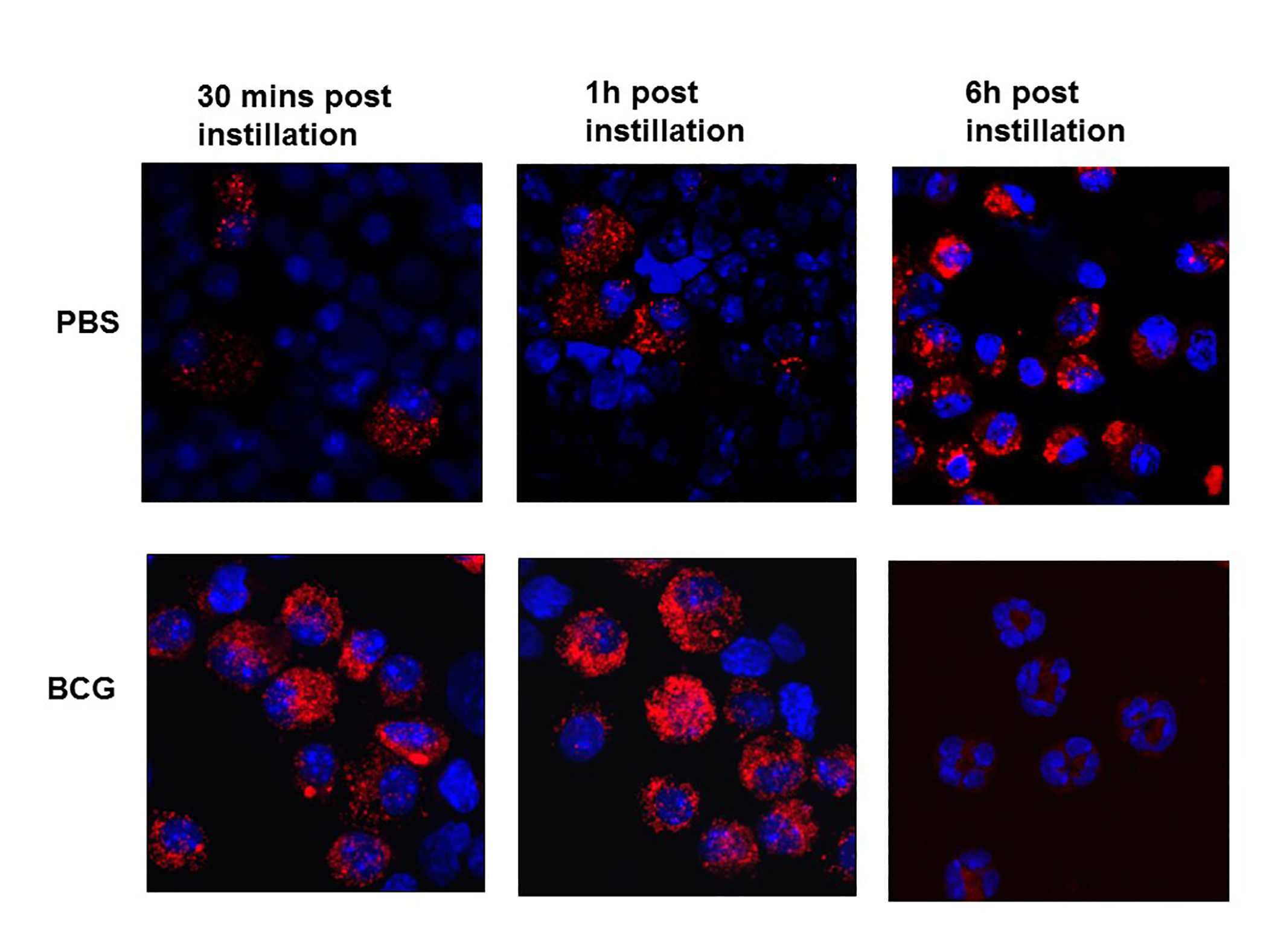

Supplement: S2 Fig — WT mice were treated as in Fig 1D. Apoptotic MLE cells were stained with red pHrodo® and administered intranasally to PBS-treated (upper row) control and BCG-treated (lower row). BAL cells were collected in different time points and subjected to confocal microscopy. 60x Magnification. Pool of BALs from 3–4 mice per group is depicted. (TIF) [file pone.0180143.s002.tif]

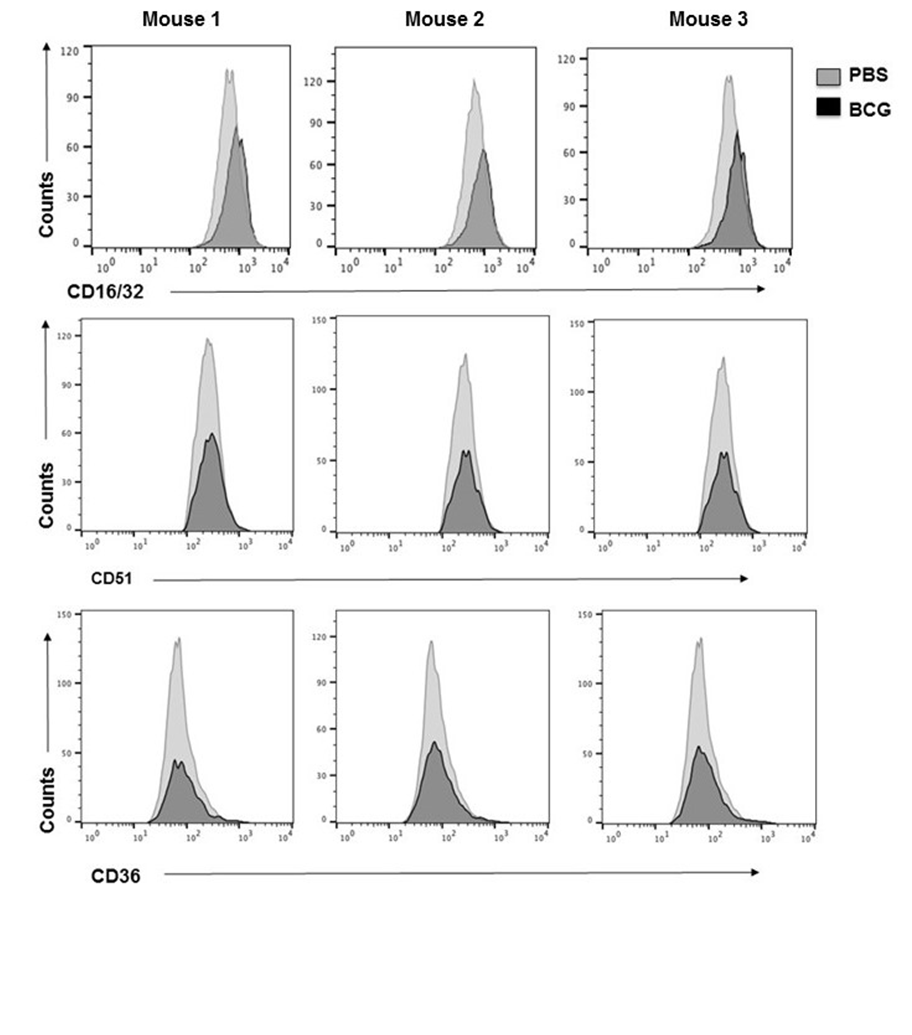

Supplement: S3 Fig — Expression of CD51, CD36, and CD16/32 on AMs of BCG- and PBS-treated control mice were analyzed by flow cytometry. Representative histograms of 3 individual mice for each group gated for F4/80 and respective markers are shown. Isotype antibodies showed baseline staining and were excluded for clarifying the effects of BCG. (TIF) [file pone.0180143.s003.tif]
